# Experiments on Cu-isotope fractionation between chlorine-bearing fluid and silicate magma: implications for fluid exsolution and porphyry Cu deposits

**DOI:** 10.1093/nsr/nwz221

**Published:** 2020-01-02

**Authors:** Haihao Guo, Ying Xia, Ruixia Bai, Xingchao Zhang, Fang Huang

**Affiliations:** 1 Bayerisches Geoinstitut, University of Bayreuth, Bayreuth 95440, Germany; 2 Department of Earth and Planetary Sciences, McGill University, Montreal H3A 0E8, Canada; 3 CAS Key Laboratory of Crust-Mantle Materials and Environments, School of Earth and Space Sciences, University of Science and Technology of China, Hefei 230026, China; 4 CAS Center for Excellence in Comparative Planetology, Hefei 230026, China

**Keywords:** Cu isotopes, cold-seal pressure vessels, aqueous fluids, silicate magma, porphyry deposit

## Abstract

Hydrothermal fluid is essential for transporting metals in the crust and mantle. To explore the potential of Cu isotopes as a tracer of hydrothermal-fluid activity, Cu-isotope fractionation factors between Cl-bearing aqueous fluids and silicate magmas (andesite, dacite, rhyolite dacite, rhyolite and haplogranite) were experimentally calibrated. Fluids containing 1.75–14 wt.% Cl were mixed together with rock powders in Au_95_Cu_5_ alloy capsules, which were equilibrated in cold-seal pressure vessels for 5–13 days at 800–850°C and 2 kbar. The elemental and Cu-isotopic compositions of the recovered aqueous fluid and solid phases were analyzed by (LA-) ICP–MS and multi-collector inductively coupled plasma mass spectrometry, respectively. Our experimental results show that the fluid phases are consistently enriched in heavy Cu isotope (^65^Cu) relative to the coexisting silicates. The Cu-isotope fractionation factor (Δ^65^Cu_FLUID-MELT_) ranges from 0.08 ± 0.01‰ to 0.69 ± 0.02‰. The experimental results show that the Cu-isotopic fractionation factors between aqueous fluids and silicates strongly depend on the Cu speciation in the fluids (e.g. CuCl(H_2_O), CuCl_2_^–^ and CuCl_3_^2−^) and silicate melts (CuO_1/2_), suggesting that the exsolved fluids may have higher δ^65^Cu than the residual magmas. Our results suggest the elevated δ^65^Cu values in Cu-enriched rocks could be produced by addition of aqueous fluids exsolved from magmas. Together with previous studies on Cu isotopes in the brine and vapor phases of porphyry deposits, our results are helpful for better understanding Cu-mineralization processes.

## Teaser

Aqueous fluids are enriched in heavy Cu isotope (higher d65Cu) relative to the silicate magmas evidenced by high temperature and pressure experiments, revealing the Cu isotopic anomaly in water-rock reaction and Cu mineralization.

## INTRODUCTION

Magmatic-hydrothermal fluids exsolved from magma play essential roles in triggering volcanic eruptions, volatile extraction, transporting metals (i.e. ‘volatile flux’) from the magmas and forming ore deposits [1]. Such aqueous fluids can be enriched in volatile and metal elements, as controlled by the partitioning behavior of these elements between the aqueous fluids and magma [2–5]. Therefore, knowledge of the composition, transportation and evolution of such fluids is critical for tracing fluid activity, volatile-related magma differentiation and understanding ore-forming element behaviors in the crust [6].

Copper is a fluid-mobile and chalcophile element with two stable isotopes (^63^Cu and ^65^Cu). Studies on coexisting fluid and silicate melt inclusions in natural samples [2,3] and experimental products [4,5] reveal that the partition coefficients of Cu between fluid and silicate melt or magma (D_FLUID/SILICATE_) are higher than 1 and mostly at a level of 10. Fumarolic gases or aerosols compositions of volcanoes from Erta Ale [7] suggest that fluids have a large capacity to scavenge and transport Cu from silicate magmas. These together suggest that magmatic fluids widely occur as critical transporting media for Cu, which can explain the anomalously high concentrations of Cu in melt inclusions at Mount Etna, Italy [8] and amphibole phenocrysts from Mount St. Helens, Washington [9], as the imprint of a Cu-rich fluid phase exsolved from magma intrusions.

With the application of multi-collector inductively coupled plasma mass spectrometry (MC–ICP–MS), Cu-isotopic compositions, expressed as δ^65^Cu (normalized ^65^Cu/^63^Cu relative to international standard NIST976 in ‰ unit), of natural samples can be precisely determined. Significant variations of δ^65^Cu have been observed in natural samples. Primary high-temperature Cu-sulfides, secondary low-temperature Cu-sulfides (and Cu-oxides) as well as Fe-oxides in the leach cap from nine porphyry copper deposits have δ^65^Cu ranging from –16.96‰ to 9.98‰ [10]. The δ^65^Cu of aqueous copper in leaching fluids and chalcocite/chalcopyrite in the supergene environment ranges from 0.58‰ to 5.59‰ [11]. Copper-isotope data have also been applied to trace fluid pathways in magmatic-hydrothermal systems [10,12]. For instance, a large Cu-isotope fractionation in chalcopyrite from successive intrusions (i.e. Grasberg, Indonesia [13]; δ^65^Cu ranges from 0.02‰ to 1.34‰) and porphyry mineralizing processes (i.e. Northparkes porphyry Cu–Au deposit, NSW, Australia [12]; δ^65^Cu from up to 0.8‰ to a low of ∼ –0.4‰) have been observed, suggesting that Cu isotopes can also be dramatically fractionated at temperatures >200°C. A large range of δ^65^Cu (–0.03‰ to 1.44‰) was reported in primary chalcopyrite from modern seafloor ‘black smoker’ chimneys, likely due to relatively rapid fluctuations of vapor content in the vent fluids [14]. Clearly, Cu isotopes have great potential in deciphering Cu transport during magmatic-hydrothermal processes.

In order to apply Cu-isotope fractionations to study magmatic-hydrothermal systems, equilibrium fractionation factors of Cu isotopes (Δ^65^Cu_FLUID-MELT_, defined as the difference in δ^65^Cu of two phases in equilibrium, i.e. fluid and magma) are required. Quantum chemical calculations suggested that volcanic degassing could lead to significant Cu-isotope fractionation between liquid and vapor phases, with ^65^Cu being preferentially concentrated in the vapor phase (Cu_3_Cl_3_) and ^63^Cu into the liquid phase ([Cu(HS)_2_]^−^ and [CuCl_2_]^−^) [15]. Copper-isotope fractionation in the CuCl–NaCl–H_2_O system have been studied, showing that ^65^Cu is enriched in the liquid relative to the vapor [16]. The discrepancies of copper fractionation results in studies [15] and [16] remain to be explored. Based on hybrid density functional simulation, Cu-isotopic fractionation was calculated during oxidation/reduction and aqueous complexation processes [17], suggesting that redox reactions can enhance significant Cu-isotope fractionation. Although these studies shed important light on the potential of Cu isotopes as a tracer for fluid activities, a Cu-isotope fractionation factor between the fluid phase and silicate magma is still absent. This hinders our understanding of Cu-isotope data of ore deposits and fluid-related igneous rocks.

In this study, we experimentally determined the equilibrium fractionation factors of Cu isotopes between hydrothermal fluids and silicate magma. The experiments were performed using cold-seal pressure vessels at 800–850°C and 2 kbar for 5–13 days. Because Cl is the major anion forming complex species with the metals in most crustal and magmatic fluids [5,18], the starting materials include Cl-bearing fluids and natural and synthesized rock powders (United States Geological Survey (USGS) rock standards and natural obsidian in Supplementary Table 1), which makes our experimental results applicable to natural systems. We measured Cu-isotope compositions for the experimental products including fluids and quenched silicate materials that have reached thermodynamic equilibrium. Our results are helpful for understanding Cu transporting via fluids in magma chambers and the formation of porphyry Cu-ore deposits.

## RESULTS

### Texture of run products

**Figure 1.**
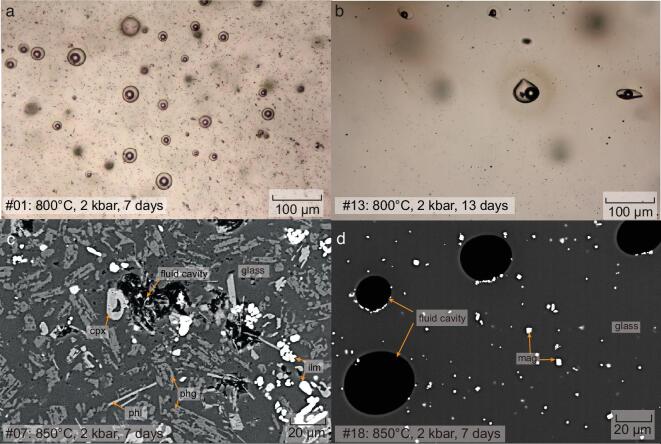
Run product photographs of the experiments. (a) Transmitted-light photomicrograph of RGM-1 from run #01 at 800°C, 2 kbar after 7 days, showing numerous bubbles in the glass. (b) Transmitted-light photomicrograph of haplogranite from run #13 at 800°C, 2 kbar after 13 days. (c) Backscattered electron (BSE) image of AGV-1 from run #07 after a run time of 7 days at 850°C and 2 kbar. (d) BSE image of obsidian from run #18 after a run time of 7 days at 850°C and 2 kbar. The vapor bubbles and solid phases in the fluid inclusions were formed by cooling during quenching process. cpx, clinopyroxene; plg, plagioclase; phl, phlogopite; mag, magnetite; ilm, ilmentite.

The solid phases, i.e. quenched magmas containing minerals and quenched glasses, show various degrees of crystallization during the high-temperature period or quenching. The corresponding mineral assemblages are listed in Supplementary Table 2 and the representative textures are shown in Fig. 1. The solid phases contain a fluid phase (fluid inclusions) or fluid cavities that represent aqueous fluids trapped in quenched silicate melt (Fig. 1), indicating the coexistence of fluid and melt during experiments. The felsic run products are homogeneous with only magnetite of 1–2 μm in RGM-1 (Fig. 1a) and there is no crystallization in haplogranite glasses (Fig. 1b). The relatively mafic run products (andesite in #07) are highly crystallized, consisting of various mineral phases (50–70 vol% depending on the starting compositions and experimental conditions) and the quenched silicate melt with cavities filled with fluids (Fig. 1c). The mineral assemblages in the various run products include clinopyroxene, plagioclase, phlogopite, magnetite, ilmenite, spinel and zircon (details in Supplementary Table 2; Fig. 1c and d). The relatively large minerals (>10 μm) and homogeneous glasses (Fig. 1c and d) in the solid phases suggest that these minerals were stable under experimental conditions, not resulting from the quenching process.

### Chemical composition of run products

**Figure 2.**
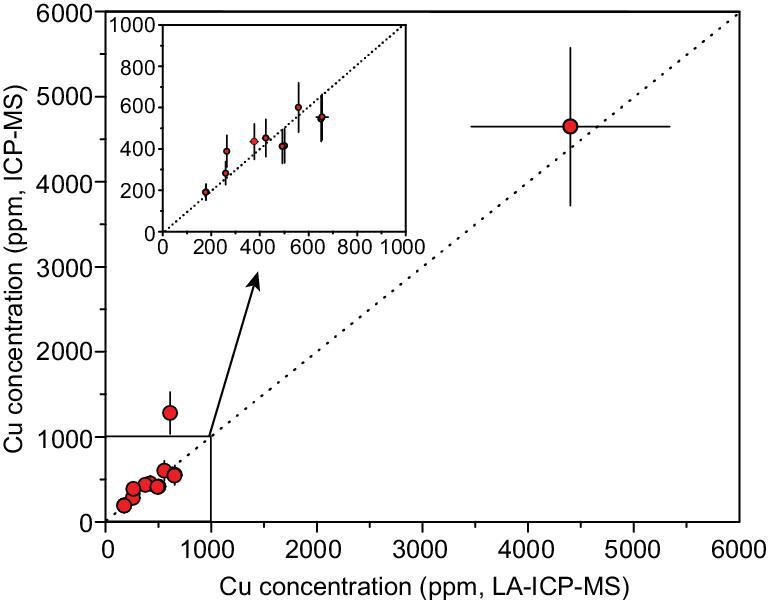
The Cu concentrations in the rock products measured by LA–ICP–MS vs. solution ICP–MS. The 1:1 correlation between the results from the two methods demonstrates that the leaching procedure avoided the contamination of fluid precipitations in the fluid cavities or fluid inclusions.

The major and trace-element concentrations in the magmas from the LA–ICP–MS measurement are reported in Supplementary Table 3. The detailed element concentrations in the fluid and solid phases measured by the solution ICP–MS method are reported in Supplementary Table 4. The Cu concentrations in solid phases (processed by leaching and acid dissolution) analyzed by solution ICP–MS and in quenched magmas (solid phases) analyzed by *in situ* LA–ICP–MS are consistent within errors (Fig. 2). It demonstrates that the solid phase is not significantly contaminated by Cu-rich fluid (as the form of fluid inclusions or cavities) because it was effectively removed by the leaching process.

**Figure 3.**
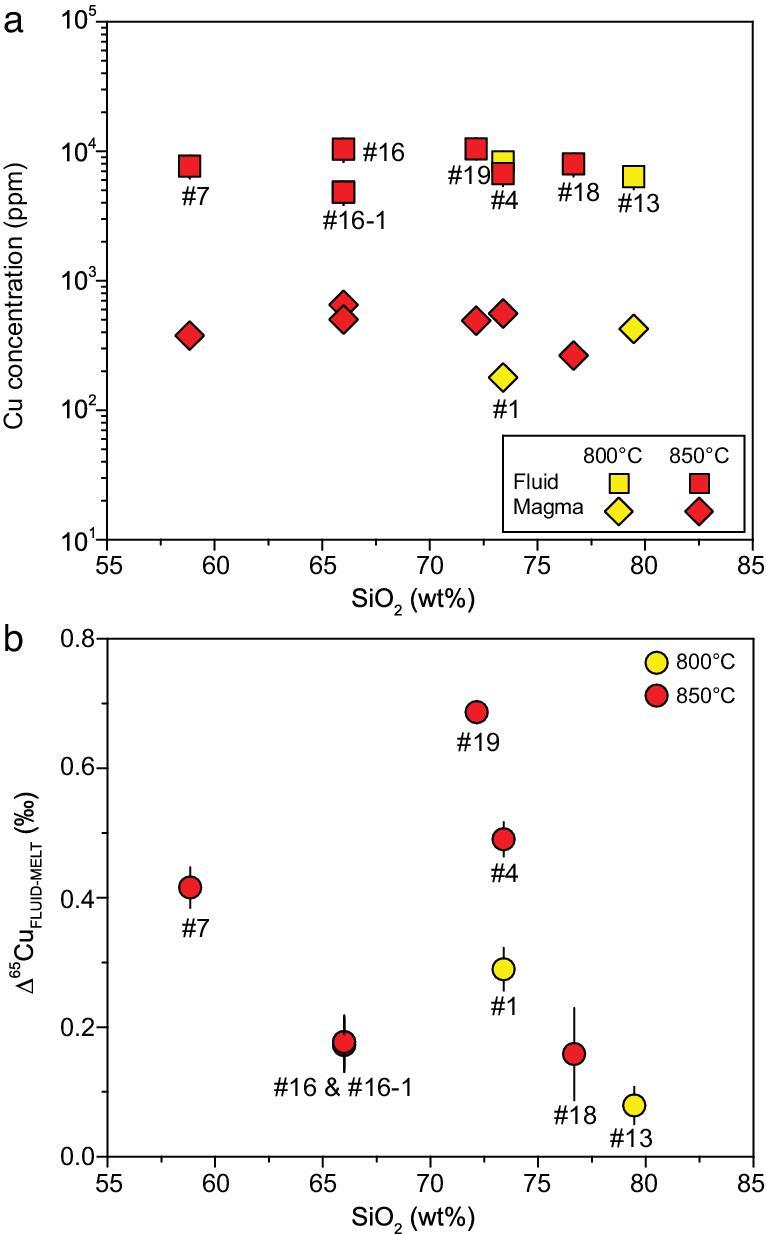
(a) The Cu concentrations in the fluid with 3.5 wt.% Cl and magmas at different temperatures. (b) The Δ^65^Cu_FLUID-MELT_ as a function of the SiO_2_ contents in magmas. Notice the data of #16 and #16–1 are overlapped in (b).

**Figure 4.**
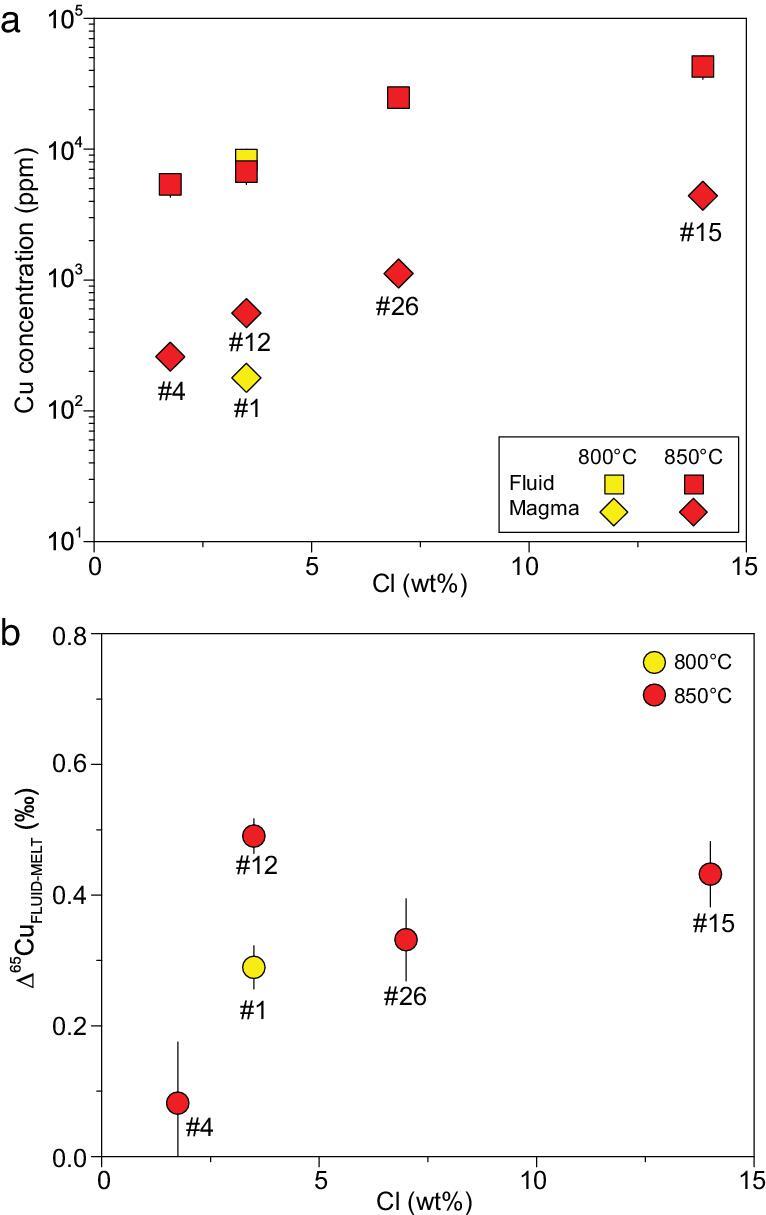
(a) The Cu concentrations in the fluid with various Cl concentrations and RGM-1 as the starting material. (b) The fluid–magma ^65^Cu fractionation factor as a function of Cl concentrations in the fluid with RGM-1 as the starting material.

In all experiments with 3.5 wt.% Cl in the fluids, Cu concentrations in fluids and solid phases are shown in Fig. 3a at various SiO_2_ contents in the starting materials, i.e. from andesite to haplogranite. The solid phases contain Cu from 192 to 601 ppm, and the fluids have much higher Cu contents from 4820 to 15440 ppm (Fig. 4a; Supplementary Table 4), demonstrating that the D_FLUID/MELT_ are around 10, in agreement with previous studies [4,5]. There is no apparent correlation between magma composition (e.g. SiO_2_ content) and Cu concentration (or solubility) in solid phase or fluids (Fig. 3a; Supplementary Table 4) if the temperature and Cl content are constant.

In the rhyolite experiments at 850°C (runs #04, 12, 26 and 15), Cu contents increase from 283 to 2650 ppm in the solid phase and from 5380 to 42700 ppm in the fluids with increasing Cl from 1.75 to 14 wt.% in the fluids (Fig. 4a; Supplementary Table 4). It indicates that increasing Cl contents in the starting materials significantly enhances the solubility of Cu in both melts and fluids, in agreement with previous studies [19–21].

### Copper-isotope composition of run products

The Cu-isotopic compositions (δ^65^Cu) in the fluids and solid phases (quenched magmas) are listed in Supplementary Table 2 and Δ^65^Cu_FLUID-MELT_ are shown in Figs. 3b and 4b. For all the experiments, the fluids have apparently higher δ^65^Cu than the coexisting magmas, suggesting significant Cu-isotope fractionation between the coexisting fluids and magmas. Δ^65^Cu_FLUID-MELT_ is independent of the rock compositions (Fig. 3b) and has no clear correlation with Cl concentrations in the fluids (Fig. 4b). Notably, for the rhyolite experiments with 3.5 wt.% Cl, the magnitude of fractionation at 800°C (0.29‰ ± 0.04‰) is slightly smaller than that at 850°C (0.49‰ ± 0.03‰) (runs #01 and 12; Fig. 4b; Supplementary Table 2). It is worth noting that Δ^65^Cu_FLUID-MELT_ value in the haplogranite experiment (run #13) reflects Cu-isotope fractionation between fluids and pure silicate melts, clearly showing preferential extraction of isotopically heavy Cu into the fluid phase (Fig. 3b; Supplementary Table 2). In experiments with other starting materials (andesite, dacite, rhyolitic dacite and rhyolite) where minerals coexist with melts under experimental conditions, the Δ^65^Cu_FLUID-MELT_ obtained here reflects the integrated effect of fluid–mineral and fluid–melt fractionation because the recovered solid phases (quenched magmas) are mixtures of silicate melt and mineral assemblage.

## DISCUSSION

### Approach to isotopic equilibrium

Demonstration of isotopic equilibrium is necessary to ensure the reliability of the experiments. Δ^65^Cu_FLUID-MELT_ in all experiments with different Au–Cu alloy capsules are always positive regardless of the significant variations of δ^65^Cu in the run products. These rule out the kinetic effects that light ^63^Cu has the tendency to be enriched in the vapor or liquid. By using Au_95_Cu_05_ alloy capsules to provide the Cu source for all the experiments, Cu activities in the fluid and melt were buffered by the capsule alloy into a constant value (Supplementary Figs 1 and 2; Supplementary Tables 5 and 6). Compared to the case using Cu-free capsules, Cu dissolution from alloy capsules into the samples would avoid continuous Cu loss during the experiments. The high fluid/rock ratios in the starting material are helpful to promote equilibrium during the experiments because Cu diffusion is rapid in fluids and melts at high temperature [22].

Several lines of evidence demonstrate that equilibrium in respect to both elements and isotopes between the fluid and silicate phases were obtained in our experiments. (i) The trace-element concentrations are relatively homogeneous along the measured profile of the solid phases with or without mineral crystallization. No clear elemental zonation is detected in the solid phases from core to rim as examined by LA–ICPMS. (ii) The durations of our experiments are similar to or longer than the durations of the experiments with similar P–T conditions that were proved to achieve equilibrium [5,6]. (iii) Time-series experiments (runs #16 for 7 days and #16-1 for 10 days) have identical Δ^65^Cu_FLUID-MELT_ within error (0.17‰ ± 0.05‰ vs. 0.18‰ ± 0.06‰; Supplementary Table 2; Fig. 3b), only slightly different with 0.08‰ ± 0.07‰ in run #22 (5 days). This demonstrates that equilibrium was attained with the duration of 7 days. (iv) Consistently positive Cu-isotope fractionation between the fluid and silicate magma phase was attained in two Au_95_Cu_5_ capsules with distinct δ^65^Cu (–0.54‰ ± 0.02‰ for the capsules from Wieland Edelwetalle, Germany and 0.29‰ ± 0.04‰ for the capsules from Sino-Platinum Metals Corp. Ltd., China; Supplementary Table 6). (v) Copper diffusivity data were used to assess whether equilibrium can be reached in the experiments. According to the experimental data [23,24], Cu diffusivity in hydrous melt under our conditions is not lower than 50 μm^2^/s. For the recovered melt with ∼2-mm diameter, the diffusion timescale is ∼0.93 days, which is much shorter that the durations (5–13 days) in our experiments. Therefore, Cu concentration and isotope gradients in the melt are negligible and isotopic equilibrium should also be reached.

### Factors controlling Cu-isotope fractionation (Δ^65^Cu_FLUID-MELT_)

The result on the haplogranite experiment (run #13) demonstrates that fluids have heavier Cu-isotopic composition than the coexisting silicate melt. Copper-isotope fractionations in the experiments containing residue minerals and melts suggest fluids may be richer in ^65^Cu than the coexisting minerals, such as clinopyroxene, plagioclase, phlogopite and magnetite. Previous studies determined that Cu-partition coefficients between silicate minerals and melts (D_mineral/melt_) are 0.04–0.45 for clinopyroxene, 0.02–0.12 for plagioclase, 0.18–1.83 for magnetite, titanomagnetite and Cr-spinel [25,26]. The bulk partitioning coefficient is around 0.2 during fractional crystallization or partial melting of the sulfide-free magmas at deep levels of crust [26]. Although our experimental conditions are different from the studies above, the mineral assemblages are among the phases in the experiments [25,26]. Therefore, Cu is incompatible in these minerals and the Cu-isotopic fractionation in this study should mainly reflect the fractionation between fluids and silicate melt.

Copper has three oxidation states: Cu(0), Cu(I) and Cu(II), in the natural systems. Copper(II) is stable only in highly oxidizing conditions found in near-surface environments [27], whereas Cu(I) occurs as the most common oxidation state in crustal hydrothermal fluids [18,21,28,29]. Experiments on Cu partitioning and solubility demonstrate that the dominant Cu speciation in the silicate melt is CuO_1/2_ at low to moderate oxidized conditions [19,20,25,26]. Copper(I)-chloride complexes under hydrothermal conditions are relatively well studied compared with other aqueous metal complexes. CuCl(H_2_O), CuCl_3_^2−^ and CuCl_2_^–^ are the predominant species in high-temperature chloride fluid, as demonstrated by X-ray absorption spectroscopy (XAS) studies or/and *ab initio* molecular simulations [18]. In the diluted chloride solutions, CuCl(H_2_O) with a linear structure is stable, while CuCl_3_^2−^ with a trigonal planar structure becomes stable in concentrated chloride solutions [18]. In addition, theoretical calculations show that the logarithm of the reduced partition function (lnβ) of the Cu(I) species in aqueous solution follow the order of enrichment in heavy Cu isotope (^65^Cu) as CuCl(H_2_O) > CuCl_2_^–^ > CuCl_3_^2−^ [17,30,31].

The Cu speciation in the fluid phase and the silicate melt phase is probably dominated by the Cu-chlorine complexes (CuCl(H_2_O), CuCl_2_^–^ and CuCl_3_^2−^) and CuO_1/2_, respectively. Thus, the Cu-isotope fractionations between the fluid phase and the melt phase should be due to different Cu speciation in the two phases. As heavy Cu isotope (^65^Cu) is always enriched in fluid phase despite the starting-material composition, chlorine concentrations and temperature differences in all the experiments, we can conclude that the ^65^Cu favors the CuCl(H_2_O), CuCl_3_^2−^ and CuCl_2_^–^ in the fluids and the ^63^Cu prefers the silicate phases.

### Monitoring volatile fluxing in magma chambers

Natural magmas may obtain or lose significant amounts of volatiles (H_2_O, CO_2_, sulfur and possibly halogens) during magmatism under various pressure and temperature conditions [32,33]. Magma chambers may experience fluid saturation and exsolution during magma decompression, crystallization and/or magma mixing. The fluids exsolved from the magmas are enriched in volatiles and metals, and they may rise and interact with overlying magmas. This phenomenon is called ‘volatile fluxing’, which has been identified by the highly volatile and metal concentrations observed in volcanic plume particles and fumarolic condensates [19,34,35]. Anomalously high concentrations of Cu in melt inclusions and silicate minerals were also used as monitors for volatile fluxing [8,9]. The higher Cu and other chalcophile element concentrations of the sulfides in rhyolite or dacite than in contemporaneous mafic magmas were interpreted as the result of transportation of sulfur-rich fluids exsolved from the underplating mafic magmas [36]. However, because Cu is highly volatile and strongly affiliated to sulfide [37,38], the majority of Cu in sulfide-bearing magmas is hosted in either monosulfide solid solution, intermediate solid solution or sulfide liquid. Thus, it is difficult to solely use Cu contents as a reliable monitor for volatile fluxing [5].

Copper-isotope fractionations are sensitive to fluid activity. As described in our experimental results, all Δ^65^Cu_FLUID-MELT_ for the fluid- and solid-phase pairs are positive. For simplifying and avoiding sulfur alloying with Au_95_Cu_5_ capsules at high temperature, our experiments focused on the sulfur-free fluid. Because Cl^−^ and HS^−^ have similar atomic mass and electronegativity, they should have similar bond energies and influence on equilibrium isotope fractionation [15]. Furthermore, because Cu can form sulfide complexes (e.g. Cu(HS)_2_^−^, CuHS) or mixed ligand species (e.g. [ClCuHS]^−^) in the hydrothermal fluid [21,28,29,39], our results for Cu-isotopic fractionation can also be applied to the fluid systems in which Cu is complexed by sulfide (e.g. HS^−^ ligand).

To quantify the Cu-isotope fractionation during fluid exsolution from magmas, we simulate such a process by Rayleigh fractionation. The isotope fractionation factor (α) is defined as

(1) \begin{equation*} \alpha = {R_{\mathit{products}}}/{R_{\mathit{reactants}}}, \end{equation*}

where *R_products_* refers to the ^65^Cu/^63^Cu in the fluids and *R_reactants_* is the ^65^Cu/^63^Cu in the volatile bearing magmas. Then, δ^65^Cu of residue magmas can be described as

(2) \begin{eqnarray*} {\delta ^{65}}\ C{u_{\mathit{magma}}} &=& \left( {{\delta ^{65}}C{u_{\mathit{initial}}} + 1000} \right)\nonumber\\ && \times\, {f^{\left( {\alpha - 1} \right)}}- 1000. \end{eqnarray*}

The instantaneous and cumulative Cu-isotope composition (δ^65^Cu) in the fluids equilibrated with the residual magmas in closed systems can be described by Equations (3) and (4):

(3) \begin{equation*} {\delta ^{65}}Cu = \left( {{\delta ^{65}}C{u_{\mathit{initial}}} + 1000} \right)\alpha \ {f^{\left( {\alpha - 1} \right)}} - 1000, \end{equation*}

(4) \begin{eqnarray*} {\delta ^{65}}Cu &=& \left( {{\delta ^{65}}C{u_{\mathit{initial}}} + 1000} \right)\nonumber\\ &&\times \left({{f^\alpha } - 1} \right)/\left({f - 1} \right) - 1000. \end{eqnarray*}

**Figure 5.**
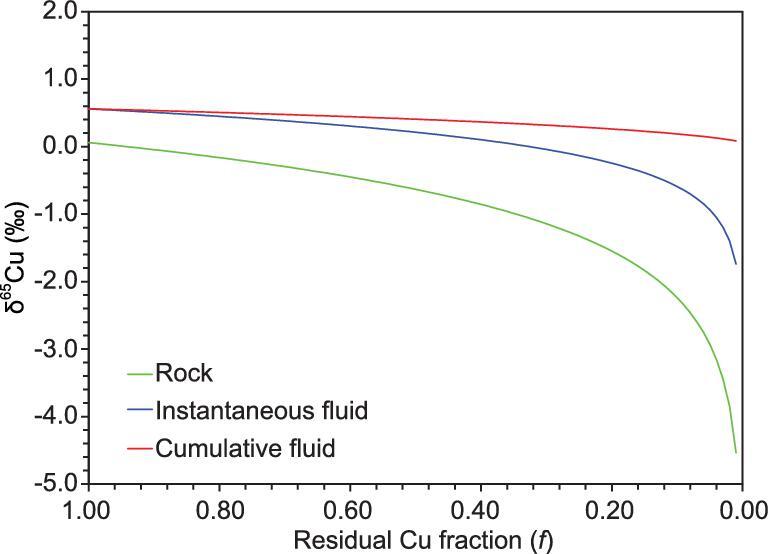
An illustration of the Cu-isotope changes in the residue magma, instantaneous and cumulative extracted fluids by Rayleigh fractionation, assuming a constant fraction factor of 1.001 (α_fluid-magma_) and an initial isotope composition of 0‰. As the fluid always extracts a significant amount of Cu from the magmas, the instantaneous and cumulative exsolved fluids have distinct δ^65^Cu, which is higher than that of the fluid-bearing magmas.

In Equations (2)–(4), δ^65^Cu_initial_ refers to the Cu-isotopic composition of the initial fluid-rich magmas and *f* is the fraction of Cu in residual magmas. As the fluid can efficiently extract Cu from magma as estimated by the partition coefficients of Cu between fluid and silicate magma (D_fluid/magma_ > 10), the fraction of Cu in residual magmas (*f*) can be lower than 0.1. The δ^65^Cu of instantaneous and cumulative fluids is significantly higher than that of residue magmas based on our numerical model (Fig. 5).

Because the fluids equilibrated with the magmas with various compositions are characterized by enrichment of the heavy Cu isotope, the higher δ^65^Cu can be regarded as an indicator for volatile addition, which may be recorded in the melt, magmatic minerals, such as sulfides and apatites precipitated from these fluids. In addition, the Cu-isotope fractionation between fluids and rocks may also be applied to indicate the fluid-flow path in the natural samples. The metasomatized peridotites have highly variable Cu concentrations and isotopic compositions (−0.64‰ to 1.82‰) compared to the non-metasomatized peridotites [40], which is possibly due to the fluid reaction with the rocks at high temperature. The fluid can scavenge heavy Cu isotope by reaction with the metasomatized peridotites, resulting in isotope fractionation relative to the primary non-metasomatized ones. On the contrary, mineral precipitation from previously ^65^Cu-rich fluids may enhance the δ^65^Cu of the peridotites [40]. Moreover, arc basalts (δ^65^Cu from −0.19‰ to 0.47‰) with higher Ba/Nb ratios [40] tend to show larger variations in δ^65^Cu, suggesting that the slab fluid is the most likely reason for the large Cu-isotopic heterogeneity in arc lavas. The large variations of δ^65^Cu in altered oceanic crusts (–0.50‰ to 0.90‰) [41] and altered abyssal peridotites on the seafloor [42] suggest that hydrothermal alteration of oceanic crust can result in significant Cu-isotope fractionation. Our study shows that the fractionation between fluids and silicate rocks is important for understanding the Cu-isotopic heterogeneity in the natural samples.

### Implications for porphyry Cu-ore deposits forming

**Figure 6.**
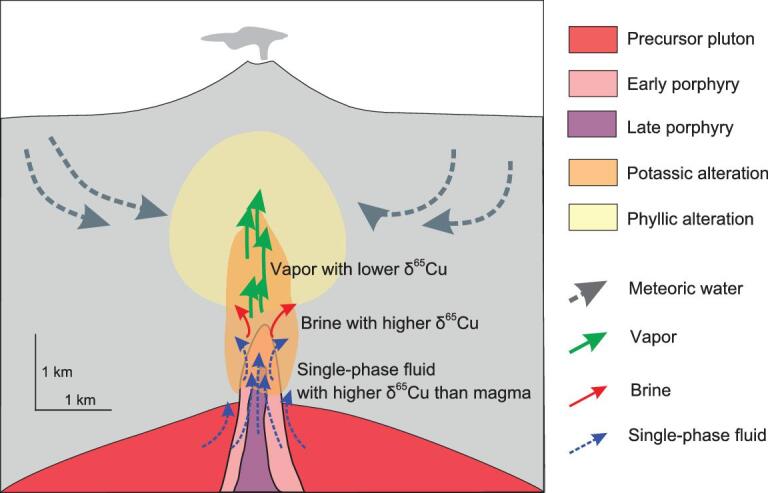
Schematic cross section of a typical porphyry Cu ± Au ± Mo system showing metal zoning, alteration patterns, fluid paths and Cu-isotope features in all types of fluids. The legend indicates the rock types, alteration zones and fluid-flow paths. The cartoon was modified from [42,52].

Porphyry Cu systems observed at the shallow part of the upper crust are commonly connected to parental magma chambers situated at 5- to 15-km depths (Fig. 6) [43]. These parental magmas should be hydrous, oxidized and sulfur-rich, in order to enhance the metal abundance in the exolved fluid phase [44]. Assemblages of coexisting fluid and melt inclusions from intrusions suggested the fluid exsolution temperature could be as high as ∼700°C [3,45]. Thus, our experimental temperatures (800–850°C) are applicable to the natural porphyry Cu-ore deposits, supposing that Cu-isotope fractionation at lower temperatures is comparable or even higher that that at higher temperatures. The porphyry Cu deposits prefer to occur in relatively oxidized conditions with oxygen fugacities of ΔFMQ = +1 to +2, where FMQ is the fayalite–magnetite–quartz oxygen buffer [46,47]. The sulfur in the aqueous fluid is dominant as SO_2_ or sulfate (SO_4_^2−^) [46,48], which does not form a complex with the Cu^+^. Our experimental redox conditions (ΔFMQ = +1.5 to +2) without sulfur are very geologically realistic for the Cu-chloride complexes in aqueous fluids of porphyry Cu-deposit systems. In the deep potassic alteration zones, porphyry Cu mineralization forms from a single-phase aqueous fluid with relatively low chlorinity (2–10 wt.% NaCl_equiv_) [2,45]. However, at the shallower part of most porphyry ore deposits, the mineralization is introduced by two-phase fluids including a small fraction of dense hypersaline liquid (brine) and a much larger fraction of low-density salt-poor fluid phase (vapor). The two phases of fluids were typically produced by decompression and cooling of a single-phase liquid, which intersects a two-phase liquid + vapor surface (V–L solvus) [32,49]. The Cu solubility and partitioning in two-phase fluids have been investigated by many natural fluid inclusion studies and experimental studies [34,50,51], revealing that the brine has a higher Cu content than the vapor. The brine generated from the deep magma is more prominent for high-grade Cu mineralization because the dense Cu-rich brine tends to remain at a depth close to the site of phase separation, while the low-density vapor may rise over the top of the porphyry intrusion.

In the porphyry Cu systems, Cu-isotope fractionation among the intruded magmas, single-phase fluid, brine and vapor are important for understanding the Cu-isotopic features in these deposits. This study depicts that the single-phase aqueous fluid has higher δ^65^Cu than the parent magmas (Fig. 6), while the vapor removed from a boiling, porphyry-type ore-forming environment has lower δ^65^Cu than the deeper brine part and the source of the ore fluids ([16] Fig. 6). These Cu-fractionation factors allow us to model the Cu-isotopic compositions in the various kinds of fluids and original magmas in porphyry deposits (Fig. 6). The single-phase fluids have significantly higher δ^65^Cu than the coexisting magmas. In addition, these fractionations may be amplified via Rayleigh fractionation in a structurally open system, in which fluids may have a progressively higher δ^65^Cu than the coexisting magmas (Fig. 6).

The experimental results can explain the observations of the Grasberg Cu–Au system [13], in which ^65^Cu is progressively enriched through the chalcopyrite grains of each successive igneous intrusion. They inferred that the Cu transport in a vapor phase enriched in ^63^Cu, resulting in higher δ^65^Cu in the residue fluid of deeper system consequent evolution with time [13]. In a study of Northparkes porphyry Cu–Au deposit, SE Australia, significant Cu-isotopic variations were observed in four drill holes [12]. δ^65^Cu decreases from around 2‰ in high-grade cores (K–feldspar, K–feldspar–biotite and biotite–magnetite alteration) of the systems to a low of ∼0.4‰ in low-grade peripheral alteration zones (phyllic–propylitic alteration zone). These patterns can be explained by fractionation of Cu isotopes among the fluids in the porphyry systems (Fig. 6). However, the explanations above only considered Cu-isotope fractionation in the single-phase fluid, brine and vapor phases.

Recently, it was reported that the fertile porphyries and mafic magmatic enclaves have elevated Cu contents and δ^65^Cu relative to barren intrusions and global-average felsic rocks [53]. Zheng *et al.* [53] suggested that Cu in the giant porphyry Cu deposits is derived from a refertilized lithosphere enriched in accumulated sulfides. Melting accumulated sulfides associated with iron oxides may contribute to economic porphyry deposits [54], which is also accompanied by further enrichment by hydrothermal fluids. According to our experimental results, fluid exsolution from magmas can further lead to the heavy Cu-isotopic signatures in these Cu-rich rocks. The partial melting from a refertilized source with high δ^65^Cu and fluid exsolution from the magmas can produce both the elevated Cu contents and heavy Cu-isotope composition in the fertile porphyries. Therefore, δ^65^Cu data in the porphyry deposits can be better utilized to fingerprint mineral exploration if the Cu-isotope fractionation mechanism in the porphyry rocks is understood by experimental studies.

## CONCLUSIONS

This study provides the first experimental study of Cu-isotope equilibrium fractionation between silicate magma and chloride-bearing fluids within a Au_95_Cu_5_ capsule in cold-seal pressure vessels under conditions relevant to natural hydrothermal fluid and silicate magma. Our results show that the fluids have ∼10 times higher Cu concentration than the coexisting magmas, with the heavy Cu isotope preferentially concentrated in fluids. Experiments with various chlorinity of fluids and composition of rock powders show a wide range of Δ^65^Cu_FLUID-MELT_ from 0.08‰ to 0.29‰ at 800 and 2 kbar, 0.08‰ to 0.69‰ at 850°C and 2 kbar. The magnitude of Cu-isotopic fractionation between fluid and magma is probably dependent on the differences in Cu speciation between fluid and silicate melt. The Cu speciation in the fluid and silicate is further controlled by fluid and rock compositions, temperature and *f*O_2_.

Our data suggest that the distinct Cu-isotope compositions in magmatic-hydrothermal fluids relative to the magmas can be used to trace natural hydrothermal-fluid activities. The exsolved fluids with higher δ^65^Cu than the residue magmas could be an indicator of the fluid exsolution and flux in magma chambers. Anomaly δ^65^Cu features in altered ocean crust and metasomatized peridotites may be due to the water–rock reaction. Together with previous studies on Cu isotopes in the brine and vapor phases of porphyry deposits [16], the results in this study can be applied to indicate fluid-flow paths by recognizing high δ^65^Cu signatures in the porphyry deposits. The elevated δ^65^Cu signatures in fertile rocks of giant porphyry Cu deposits could inherit Cu sources from a refertilized lithosphere enriched in accumulated sulfides, which could also be accompanied by fluid exsolution from the magmas. Therefore, δ^65^Cu variation is a useful indicator for the buried Cu mineralization at depth.

## METHODS

### Experimental procedure

In order to investigate Cu-isotope fractionation between fluids and magmas with various compositions, we used six glasses or rock powders with a range of compositions from felsic to andesitic as starting materials (Supplementary Table 1). AGV-1 is a natural andesite and RGM-1 is a rhyolite from the USGS geochemical reference materials (https://crustal.usgs.gov/geochemical_reference_standards/powdered_RM.html). D11 is a dacite glass and RD11 is a rhyolite dacite glass, which were synthesized in [55]. The other two felsic materials are a haplogranite of 1-kbar eutectic melt composition and a natural obsidian from China [5]. The fluids contain 1.75–14 wt.% HCl, which were prepared by mixing the analytical concentrated HCl (32 wt%) with deionized water (Supplementary Table 2).

All experiments were conducted in Bayerisches Geoinstitut, University of Bayreuth. The capsules are Au_95_Cu_5_ with 5.0 mm O.D. (outer diameter), 4.6 mm I.D. (inner diameter) and 20 mm length from Wieland Edelwetalle (the manufacturer), Germany or 4.4 mm O.D., 4.0 mm I.D. and 25 mm length from Sino-Platinum Metals Corp. Ltd., China. The capsules served as Cu source for the samples because Cu could be quickly dissolved from the capsules into fluids and melts during the experiment; the apparent concentration of Cu in run products was controlled by the reaction between the capsule and run products. Given that the Cu mass of a capsule is usually >50 times the Cu mass of the final fluid and rock powder enclosed, the capsules should buffer the activity of Cu during the experiments and yield enough Cu in the run products for further measurement. Silicate glass or rock powder was carefully loaded into capsules with the nearly similar amount of Cl-bearing solution (∼100 mg) (Supplementary Table 2). Capsules were then welded and subjected to ∼1 kbar H_2_O pressure to check potential leaks and the capsules with obvious weight changes were discarded.

The capsules were then loaded into vertical rapid-quench cold-seal pressure vessels made of Inconel 713LC super alloy using water as the pressure medium. Temperatures were measured with NiCr–Ni (K-type) thermocouples in an external borehole of the vessels. The uncertainties in the temperature and pressure are smaller than 5°C and 30 bar, respectively. Oxygen fugacity was not specifically controlled in the vessels, but it should be 0.5–1 log unit above the Ni–NiO buffer (ΔFMQ = +1.5 to +2), as suggested by the reaction of water with the autoclave material [56]. All experiments were run at 800–850°C and 2 kbar for 5–13 days (Supplementary Table 2). The samples were quenched by dropping the external magnet to let the sample fall into the water-cooled zone within a few seconds.

The capsules were recovered and weighed again to check for potential leaks during the experiments. Those without obvious weight loss were then cleaned, cooled by liquid-N_2_ and then punctured with a steel needle. After the solution was withdrawn as much as possible with a micropipette while the aqueous fluids melted, the capsules were then opened and boiled in deionized water for 30 minutes. After that, the capsule and the silicate solid phase were further rinsed several times with deionized water. All solutions obtained during these operations were added together with the solution phases withdrawn from the experiment. This is similar to the treatment of [4] that was to redissolve materials precipitated from the fluid during quenching. Indeed, *in situ* synchrotron X-ray fluorescence studies in externally heated diamond anvil cells later showed that this method works well in relatively low-pressure experiments [57]. The capsules were then cut open using a razor blade and a portion of the recovered solid phase (∼30 mg) was polished for measurement of major and trace elements by LA–ICP–MS. As the solid run products are homogeneous, random pieces were mounted in epoxy and carefully polished with abrasive paper, followed by a series of diamond pastes. The remaining solid phase was ground to powder in a mortar with grain sizes <200 mesh, then leached with ethanol and Milli-Q H_2_O a few times to remove possible contamination and fluid trapped in the quenched glass, and finally dried down for wet chemistry analyses.

### SEM and energy-dispersive spectrometer (EDS)

Backscattered electron (BSE) images of polished sections of the recovered solid phase were taken on a Zeiss Gemini 1530 field emission scanning electron microscope (FE-SEM) at Bayerisches Geoinstitut, University of Bayreuth. The accelerating voltage was 15–20 kV. The working distance was ∼14 mm and the aperture was 60 μm. An EDS was used to qualitatively identify mineral phases in the run products.

### Major and trace-element concentrations

Major and trace-element concentrations in solid-phase products were measured by laser-ablation ICP–MS with a 193-nm Excimer Laser (Coherent, USA) attached to an Elan DRC-e (Perkin Elmer, Canada) quadrupole mass spectrometer at Bayerisches Geoinstitut, University of Bayreuth. The laser was operated at a frequency of 10 Hz with ∼10 J/cm^2^ energy density on the sample surface. The laser pit size was 50 μm and the typical measuring times were 40–50 s on background and 20–30 s on the sample. The run products were analyzed at three to six points along the profiles and averages plus standard deviations were calculated. The isotopes ^7^Li, ^11^B, ^23^Na, ^25^Mg, ^27^Al, ^30^Si, ^39^ K, ^43^Ca, ^49^Ti, ^51^ V, ^53^Cr, ^55^Mn,^57^Fe, ^59^Co, ^62^Ni, ^65^Cu, ^66^ Zn, ^69^Ga, ^85^Rb, ^88^Sr, ^90^Zr, ^93^Nb, ^98^Mo, ^133^Cs, ^137^Ba, ^140^Ce, ^178^Hf, ^181^Ta, ^184^ W, ^208^ Pb, ^232^Th and ^238^U were measured using dwell times of 10–50 ms per isotope. NIST SRM 610 glass was used as the external standard, using the preferred values listed on the GeoReM database (http://georem.mpch-mainz.gwdg.de/). Internal standardization was done by normalizing the sum of all major element oxides to 100 wt.%.

The crushed solid phase and the fluid samples were also dissolved in double-distilled, concentrated acids successively: a 3:1 mixture of HF-HNO_3_, a mixture of HCl-HNO_3_ and 1 ml HCl. A small fraction of each sample solution was diluted in 2% HNO_3_ at appropriate levels for element-composition analyses using a Perkin-Elmer ELAN DCR-II inductively coupled plasma source mass spectrometer (ICP–MS) at the CAS Key Laboratory of Crust-Mantle Materials and Environments, University of Science and Technology of China, Hefei (USTC). Wet chemical analyses were conducted for two purposes: (i) to evaluate the degree of contamination of the solid phase by fluid through comparison with the results from *in situ* analyses, (ii) to obtain the solute compositions, which is important to evaluate the controlling factors of Cu-isotope fractionation.

### Cu-isotope analyses

The fully digested samples and USGS standards were taken up in 1 ml 6 N HCl + 0.001% H_2_O_2_ for ion chromatography. After the sample was loaded onto the column in 1 ml of 6 N HCl + 0.001% H_2_O_2_, matrix elements were then eluted with 5 ml of 6 N HCl + 0.001% H_2_O_2_. The Cu was eluted in a further 26 ml of 6 N HCl + 0.001% H_2_O_2_. The whole purification procedure was repeated twice to ensure removal of most matrix elements. The yields of copper through column chemistry, obtained by analyses of Cu contents in the elution collected before and after the Cu cut, were >99.5%. Total procedural Cu blanks were <5 ng, which is insignificant relative to the amounts of Cu (1–10 μg) loaded onto columns.

The isotopic analyses were performed on a Thermo Scientific Neptune Plus MC–ICP–MS in low-resolution mode at USTC. The purified samples were dissolved in 2% HNO_3_ and introduced into the instrument using an ESI PFA microflow nebulizer with an uptake rate of 50 μL/min. The ^63^Cu- and ^65^Cu-isotope beams were collected in C and L2 faraday cups, respectively. Nickle and Zn were monitored using ^62^Ni and ^64^Zn beams in L3 and L1 in the same cup set-up.

To correct the instrumental mass bias, samples were measured using the sample-standard bracketing protocol relative to the NIST SRM 976 standard. The δ^65^Cu of each sample is the average of three analyses. Instrumental drift was monitored using two mono-elemental reference materials ERM-AE-647 and AAS. The δ^65^Cu of these two standards measured at USTC are 0.19‰ ± 0.05‰ (2 SD, *n* = 347) and 0.30‰ ± 0.05‰ (2 SD, *n* = 51), respectively, suggesting that the long-term external precision of δ^65^Cu of our data is better than 0.05‰ (2SD, 95% confidence interval). USGS rock standards (e.g. BCR–1, AGV–1, RGM–1 and BHVO–2) were purified along with the samples to test the accuracy of the Cu-isotope measurement (Supplementary Table 2).

## Supplementary Material

nwz221_Supplemental_Files
