# Supplementary material for: Experiments on Cu-isotope fractionation between chlorine-bearing fluid and silicate magma: implications for fluid exsolution and porphyry Cu deposits
Source: Natl Sci Rev. 2020 Jan 2;7(8):1319–30. doi: 10.1093/nsr/nwz221 (PMC8288860; doi:10.1093/nsr/nwz221)
Supplement: nwz221_Supplemental_Files [file nwz221_supplemental_files.zip › Supplementary_Tables.pdf]

Supplementary Table 1. Major element compositions (wt%) of starting materials.

oxide	AGV-1	D11	RD11	RGM-1	Haplogranite	Obsidian
Na_2O	4.3	5.4	3.6	4.1	3.9	4.9
MgO	1.5	1.5	0.3	0.28	0.02	0.004
Al_2O_3	17.2	18.8	16.1	13.7	11.7	11.2
SiO_2	58.8	66.0	72.2	73.4	79.5	76.7
K_2O	2.9	2.1	3.4	4.3	4.91	4.3
CaO	4.9	2.5	1.8	1.15	0.06	0.19
TiO_2	1.1	0.7	0.6	0.27	0.02	0.13
FeO_t	6.8	2.3	1.3	1.86	0.04	2.43

AGV-1 and RGM-1 are from USGS (United States Geological Survey) geochemical reference materials . D11 and RD11 are from Masotta et al. (2016). Haplogranite and obsidian are from Guo and Audétat (2017).

SupplementaryTable 2. Experimental conditions and copper isotopic compositions of run products and USGS standards.

run No.	T (°C)	P (kbar)	duration (days)	starting material	SiO_2(wt%)	phase assemblage	Cl in solution (wt%)	$\delta^{65}Cu_{\_magma}$	2SD	$\delta^{65}Cu_{\_fluid}$	2SD	$\Delta^{65}Cu_{FLUID\text{-}MELT}$	2SD
**Run products**													
#01	800	2	7	RGM	73.4	melt, mag	3.5	-0.50	0.02	-0.21	0.02	0.29	0.04
#04	850	2	7	RGM	73.4	melt, mag	1.75	-0.48	0.05	-0.40	0.04	0.08	0.10
#12	850	2	10	RGM	73.4	melt, mag	3.5	-0.53	0.00	-0.04	0.02	0.49	0.03
#26	850	2	7	RGM	73.4	melt, mag	7	-0.66	0.06	-0.33	0.01	0.33	0.06
#15	850	2	13	RGM	73.4	melt, mag	14	0.17	0.03	0.60	0.04	0.43	0.06
#13	800	2	13	haplogranite	79.5	melt	3.5	0.48	0.01	0.56	0.03	0.08	0.04
#07	850	2	7	AGV	58.8	melt,cpx, phl,plg,iml	3.5	-0.49	0.01	-0.07	0.02	0.42	0.04
#22	850	2	5	D11	66.0	melt, spl, cpx,zrn	3.5	0.28	0.04	0.36	0.03	0.08	0.07
#16	850	2	7	D11	66.0	melt, spl, cpx,zrn	3.5	0.40	0.03	0.58	0.03	0.17	0.05
#16.1	850	2	10	D11	66.0	melt, spl, cpx,zrn	3.5	0.36	0.01	0.54	0.04	0.18	0.06
#18	850	2	7	obsidian	76.7	melt, mag	3.5	0.46	0.06	0.61	0.04	0.16	0.08
#19	850	2	7	RD11	72.2	melt, mag	3.5	-0.43	0.01	0.26	0.01	0.69	0.02
**USGS standards**													
BHVO-2								0.14	0.02				
BCR-2								0.20	0.02				
RGM-1								0.05	0.01				
AGV-1								0.02	0.04				
AGV-1 duplicate								0.03	0.05				

Abbreviations: cpx - clinopyroxene; plg - plagioclase; phl - phlogopite; mag - magnetite; ilm-ilmentite; spl-spinal; zrn-zircon.

#1,4,7,12,26 were performed by the $Au_{95}Cu_5$ capsules from Wieland Edelwetalle, Germany; #13,15,16,16-1,18,19,22 were performed by the $Au_{95}Cu_5$ capsules from Sino-Platinum Metals Corp. LTD., China.

Supplementary Table 3. LA-ICP-MS results of quenched magma (solid phase product) compositions.

Sample	#1	1σ	#4	1σ	#12	1σ	#15	1σ	#26	1σ	#13	1σ	#7	1σ	#22	1σ	#16	1σ	#16-1	1σ	#18	1σ	#19	1σ
wt%																								
SiO_2	76	0.1	77	0.2	77	0.3	80.3	1.5	79.0	0.02	80	0.4	68	0.5	69.2	0.4	70	0.3	71	0.4	77	0.4	73	0.2
Al_2O_3	14.6	0.3	12.8	0.1	12.8	0.2	14.1	1.1	12.8	0.05	12.0	0.4	16.6	0.3	17.9	0.2	17.2	0.2	16.4	0.3	11.5	0.1	15.7	0.1
CaO	1.2	0.03	1.2	0.05	1.2	0.06	0.8	0.1	1.2	0.04	0.02	0.003	5.2	0.24	1.83	0.05	1.8	0.0	1.7	0.1	0.2	0.004	2.0	0.04
MgO	0.24	0.01	0.3	0.002	0.3	0.01	0.2	0.01	0.25	0.002	0.02	0.0003	1.4	0.11	1.12	0.13	1.1	0.1	0.8	0.03	0.01	#####	0.4	0.01
FeO_T	1.0	0.23	1.3	0.1	1.0	0.01	0.1	0.01	0.59	0.01	0.01	0.0005	1.8	0.1	1.2	0.1	1.4	0.1	1.3	0.01	2.5	0.3	0.9	0.02
Na_2O	3.2	0.0	3.8	0.01	3.6	0.03	1.5	0.1	3.0	0.010	3.3	0.1	3.8	0.01	5.08	0.05	5.1	0.1	5.1	0.03	4.2	0.1	3.5	0.03
K_2O	3.7	0.1	3.8	0.02	3.6	0.02	1.8	0.2	2.9	0.008	4.1	0.1	2.5	0.1	2.1	0.02	2.1	0.01	2.2	0.03	3.6	0.1	3.3	0.03
TiO_2	0.24	0.01	0.2	0.01	0.3	0.003	0.3	0.01	0.26	0.002	0.02	0.00	0.4	0.0	0.7	0.03	0.7	0.1	0.4	0.003	0.2	0.01	0.6	0.1
ppm																								
Li	50	1.1	60	1.6	57	1.2	24	2.9	51.0	1.8	3.0	0.4	10	0.7	n.a.	n.a.	40	0.6	38	1.3	33.4	1.2	38.1	0.2
B	16	6	21	3.8	23	1.9	16	0.8	21.3	1.2	22.3	1.5	5	0.5	n.a.	n.a.	2.5	1.0	2.9	0.4	35.3	1.3	2.8	0.6
V	10	1.0	11	0.7	11	0.7	10	1.2	9.9	0.4	4.1	0.2	36	4.0	0.7	0.6	0.4	0.03	0.4	0.02	0.1	0.03	0.5	0.02
Cr	23	6	<4		<3		4.8	0.6	<4		3.6	2.4	5.2	0.7	3.0	4.3	2.1	0.9	0.8	0.1	0.1	0.2	24.2	15.7
Mn	137	15	239	3.2	167	3.3	27	2.4	81.8	1.1	0.7	0.2	444	5.9	31.9	1.4	32	0.7	29	1.0	343	25	22	0.2
Co	1.6	0.7	6.8	0.8	6	0.4	3.5	0.5	7.4	0.6	2.9	0.4	11	1.1	24.6	3.5	2.1	0.4	4.3	0.2	4.8	2.8	19.8	3.5
Cu	179	11	259	4.8	558	8	4,400	940	613	2	424	15	376	16	177	8	652	5.4	502	9	264	11	492	11
Zn	57	3.8	208	11	86	1.8	29	6.8	33.0	0.8	19	2.4	215	6.7	75.1	18.7	38	19	33	2.7	142	18	58	4.0
Ga	30	4.7	52	5.4	48	0.9	7.7	1.5	52.4	1.7	7.0	0.2	84	5.8	9.4	0.8	8.3	0.8	9.4	0.2	29	1.1	11.5	3.0
Rb	120	2.8	146	1.2	134	0.8	60	7.4	109.4	0.9	1.9	0.1	61	1.3	5.0	0.1	4.9	0.1	5.3	0.05	129	3.2	5.4	0.1
Sr	101	3.8	101	2.6	101	2.8	77	6.2	101.7	0.9	112	2.3	671	22	4	0.1	3.8	0.04	4.2	0.1	0.9	0.04	7.3	0.1
Zr	202	20	202	38	241	21	200	142	237	65	9.5	0.6	193	4	5,592	851	3,120	2,500	5,500	540	835	357	5,300	1,760
Nb	8.6	0.2	9	0.1	9.3	0.0	9.5	0.7	9.3	0.3	0.5	0.02	13	0.4	0.1	0.01	0.1	0.0	0.1	0.004	65	0.4	0.1	0.03
Mo	2.0	0.4	2.3	0.1	2.1	0.2	0.3	0.1	1.8	0.2	0.1	0.1	0.7	0.05	n.a.	n.a.	1.2	0.1	1.3	0.1	9.2	1.5	0.8	0.1
Cs	7.5	0.3	9	0.2	8.1	0.1	3.3	0.3	6.9	0.2	0.1	0.02	0.9	0.03	0.15	0.14	0.2	0.01	0.2	0.005	2.8	0.1	0.2	0.02
Ba	816	30	818	9.1	827	35	670	56	827	7	17	0.4	1436	50	11	0.6	10.1	0.1	11.9	0.2	2.4	0.2	15.1	0.6
Ce	42	4.1	45	0.9	45	0.2	41	3.3	46.3	0.4	8.6	0.1	94	1.0	3.0	0.2	2.7	0.1	2.7	0.1	150	2.3	2.7	0.1
Hf	5.6	0.4	5.7	0.8	6.4	0.2	6.3	4.7	6.6	1.6	0.3	0.02	4.5	0.2	129.5	19.9	83	65	130	15	22	7.4	129	37
Ta	0.9	0.04	0.9	0.1	1.0	0.1	0.9	0.1	1.0	0.1	0.1	0.01	0.6	0.1	0.02	0.01	0.02	0.02	0.03	0.01	4.1	0.1	0.03	0.01
W	2.6	0.6	1.6	0.4	2.0	0.1	50	4.4	3.1	0.4	4.1	0.3	1.5	0.3	n.a.	n.a.	13.5	0.6	14.1	0.1	2.5	0.2	14.3	1.5
Pb	6.6	0.3	15	0.1	9.4	0.4	3.0	0.6	3.9	0.1	1.1	0.03	16	0.1	3.6	0.1	2.8	0.03	5.5	0.1	8.8	1.2	2.9	0.1
Th	14	0.6	14	0.1	14	0.4	15.3	2.8	14.7	0.6	0.7	0.03	7.4	0.2	0.1	0.0	0.04	0.03	0.05	0.01	18.2	2.3	0.1	0.02
U	5.2	0.6	5.9	0.4	5.8	0.2	4.3	1.4	5.6	0.2	0.4	0.02	2.3	0.1	0.1	0.0	0.1	0.04	0.1	0.02	7.2	1.1	0.1	0.02

n.a.- not analyzed

Supplementary Table 4. Recovery solid phase (quenched magma) and fluid compositions analyzed by ICP-MS.

Sample	#1		#4		#12		#15		#26		#13		#7		#22		#16		#16-1		#18		#19	
ppm	magma	fluid	magma	fluid	magma	fluid	magma	fluid	magma	fluid	magma	fluid	magma	fluid	magma	fluid	magma	fluid	magma	fluid	magma	fluid	magma	fluid
Li	36	14	59	11.2	52	11.7	16	41.0	2.4	0.2	2.5	1.9	9.3	6.3	27.8	9.2	34	9.0	29	5.9	28	10.0	32	3.9
Na	12,858	4,466	25,310	4,080	21,689	5,362	5,966	17,592	17,654	9,852	24,401	4,993	23,160	7,560	22,281	5,710	28,657	7,869	25,539	3,743	23,889	6,640	18,703	1,353
Mg	1587	40	1578	15	1502	27	875	527	1374	100	93	10	9091	179	7864	115	6693	103	6270	76	130	35	1688	0
Al	103,459	53	71,680	179	66,071	256	64,776	730	66,891	434	55,322	106	90,982	173	93,860	187	81,338	227	76,644	135	48,999	148	62,051	97
K	19,265	5,317	31,445	4,146	26,418	6,288	9,541	19,615	22,034	11,413	30,896	6,563	17,704	6,341	6,741	1,793	12,315	3,915	11,052	1,664	24,374	6,143	20,070	1,145
Ca	98,309	354	2,970	36	2,681	44	n.a.	n.a.	2,607	159	99	<0.3	12,138	221	15,016	437	n.a.	n.a.	n.a.	n.a.	n.a.	n.a.	n.a.	n.a.
Sc	n.a.	n.a.	4.6	n.a.	4.4	n.a.	5.0	1.7	4.1	n.a.	3.8	1.5	12.7	n.a.	n.d.	n.d.	2.2	1.5	2.1	1.2	3.8	1.8	2.7	1.7
Ti	1,034	2.9	1,009	11	1,256	7.3	1,447	31	901	8.4	129	11	5,530	10	3,969.9	6.0	3,481	25	3,128	33	681	22	2,613	17
V	13	5	3.9	n.a.	3.2	n.a.	n.a.	n.a.	n.a.	n.a.	73	50	138	n.a.	n.d.	n.d.	n.a.	n.a.	n.a.	n.a.	n.a.	n.a.	n.a.	n.a.
Cr	n.a.	n.a.	2.0	n.a.	n.a.	n.a.	6.7	1.7	n.a.	n.a.	<3	<3	6.5	n.a.	16.6	0.79	19	1.1	10	1.5	3.8	3.1	23	1.6
Mn	112	120	254	70	173	107	16	252	82	202	<4	<0.3	591	219	18	15	32	15	27	8	294	221	24	9
Fe	5,427	5,100	10,668	2,075	7,318	4,718	944	8,582	3,326	8,471	137	278	44,958	4,455	8,454	6,470	10,236	3,798	9,167	1,482	11,878	3,326	5,198	926
Co	n.a.	n.a.	6.9	5.6	7.2	11	n.a.	n.a.	4	15	6.8	36.3	19.4	5.0	47	53	n.a.	n.a.	n.a.	n.a.	n.a.	n.a.	n.a.	n.a.
Ni	2393	4493	95	48	148	167	48	1091	83	530	130	517	376	86	157	44	323	94	541	74	572	576	368	62
Cu	192	8,241	283	5,378	601	6,707	4,647	42,696	1,279	24,705	554	6,355	436	7,679	190	4,435	544	10,341	415	4,823	389	7,977	412	1,428
Zn	47	768	175	661	98	864	16	566	29	967	28	931	280	813	142	711	236	543	243	248	203	666	152	162
Rb	92	29	139	20	113	31	n.a.	n.a.	97	57	23	10.2	49	22	15	5	n.a.	n.a.	n.a.	n.a.	n.a.	n.a.	n.a.	n.a.
Sr	130	2.7	120	11	110	12	n.a.	n.a.	106	16	99	<3	754	19	6	1	n.a.	n.a.	n.a.	n.a.	n.a.	n.a.	n.a.	n.a.
Ba	770	28	887	81	824	67	565	326	804	111	23	55	1,273	101	10	56	9.0	46	10	50	2.3	63	14	55

n.a.- not analyzed

Supplementary Table 5. Copper concentrations measured by EDS for the recovered $Au_{95}Cu_5$ capsules. The units are in wt%.

Capsule No.	inner rim	middle	outer rim
#01	4.42	4.27	4.62
σ	0.29	0.28	0.3
#04	4.32	4.5	4.32
σ	0.28	0.29	0.27
#12	4.26	4.24	4.37
σ	0.28	0.28	0.28

Supplementary Table 6. Copper isotopic compositions of initial and recovered $Au_{95}Cu_5$ capsules.

Capsule No.	ini[a]	#1	#7	#12	ini[b]	#13	#16
$\delta^{65}Cu$ (‰)	-0.54	-0.52	-0.52	-0.52	0.29	0.29	0.27
2SD	0.02	0.02	0.04	0.02	0.04	0.03	0.02
N[c]	3	3	3	3	3	3	3

[a]The initial Cu isotopic composition of $Au_{95}Cu_5$ capsules from Wieland Edelwetalle, Germany.

[b]The initial Cu isotopic composition of $Au_{95}Cu_5$ capsules from Sino-Platinum Metals Corp. LTD., China.

[c]Measured times.
