# Supplementary material for: Experiments on Cu-isotope fractionation between chlorine-bearing fluid and silicate magma: implications for fluid exsolution and porphyry Cu deposits
Source: Natl Sci Rev. 2020 Jan 2;7(8):1319–30. doi: 10.1093/nsr/nwz221 (PMC8288860; doi:10.1093/nsr/nwz221)
Supplement: nwz221_Supplemental_Files [file nwz221_supplemental_files.zip › Supplementary_material.docx]

Experiments on Cu isotope fractionation between chlorine bearing fluid and silicate magma: implications for fluid exsolution and porphyry Cu deposits

Haihao Guo^a,b^, Ying Xia^c,^, Ruixia Bai^c^, Xingchao Zhang^c^, Fang Huang^c,d^

^a^Bayerisches Geoinstitut, University of Bayreuth, Bayreuth 95440, Germany

^b^Department of Earth and Planetary Sciences, McGill University, Montreal, Quebec H3A 0E8, Canada

^c^CAS Key Laboratory of Crust-Mantle Materials and Environments, School of Earth and Space Sciences, University of Science and Technology of China, Hefei 230026, China

^d^CAS Center for Excellence in Comparative Planetology, Hefei 230026, China

**Supplementary Information**

**
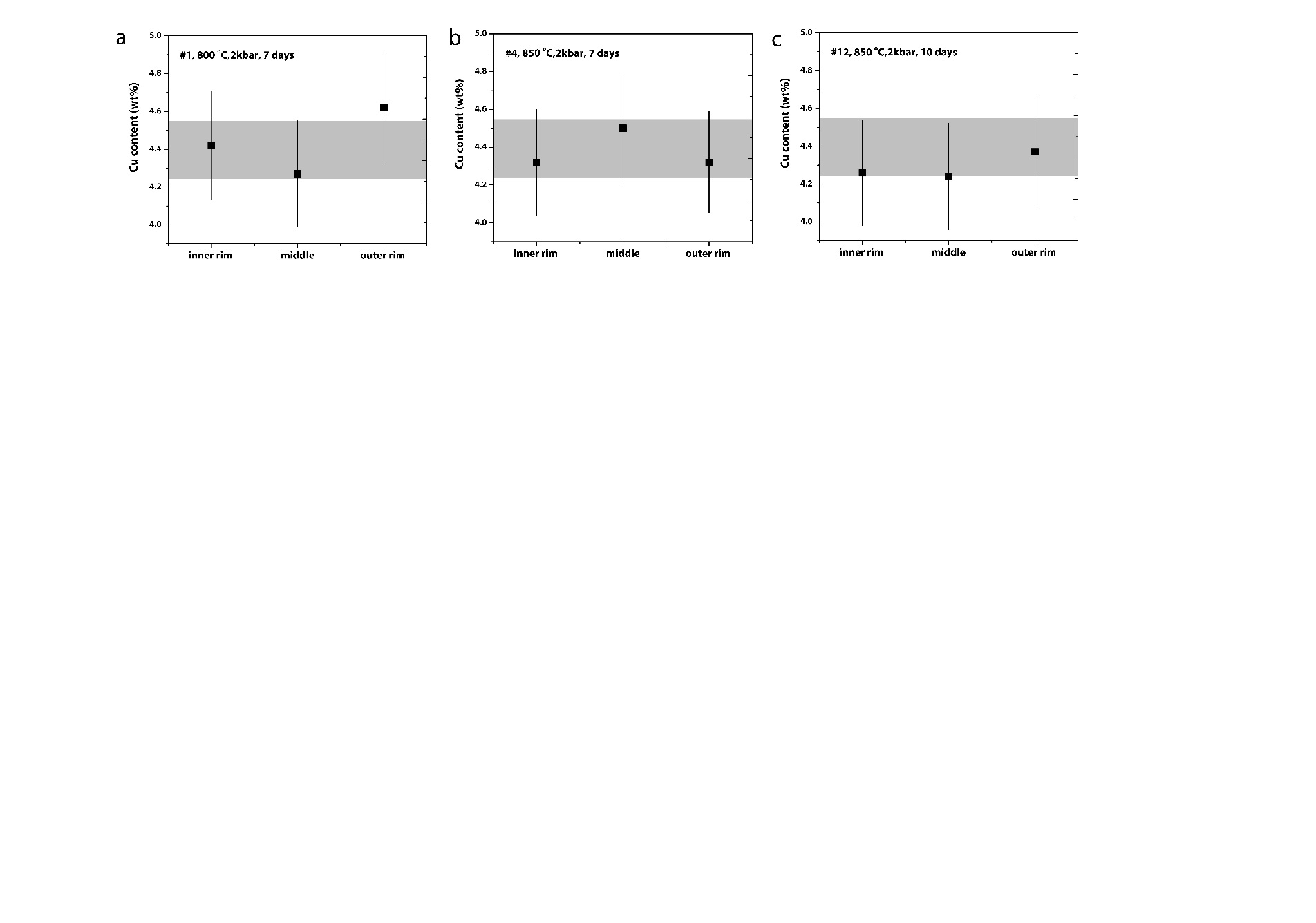
**

**Figure S1.** Cu concentration profiles for Au_95_Cu_5_ capsules from the inner rims to the outer rims. (a) run #1 at 800 °C and 2 kbar, 7 days, (b) run #4 at 850 °C and 2 kbar, 7 days and (c) run #12 at 850 °C and 2 kbar, 10days. The grey area represents the initial Cu concentration before the experiments. The results show there is no Cu zonation in the recovered capsules and the loss of Cu into the samples is negligible.

**
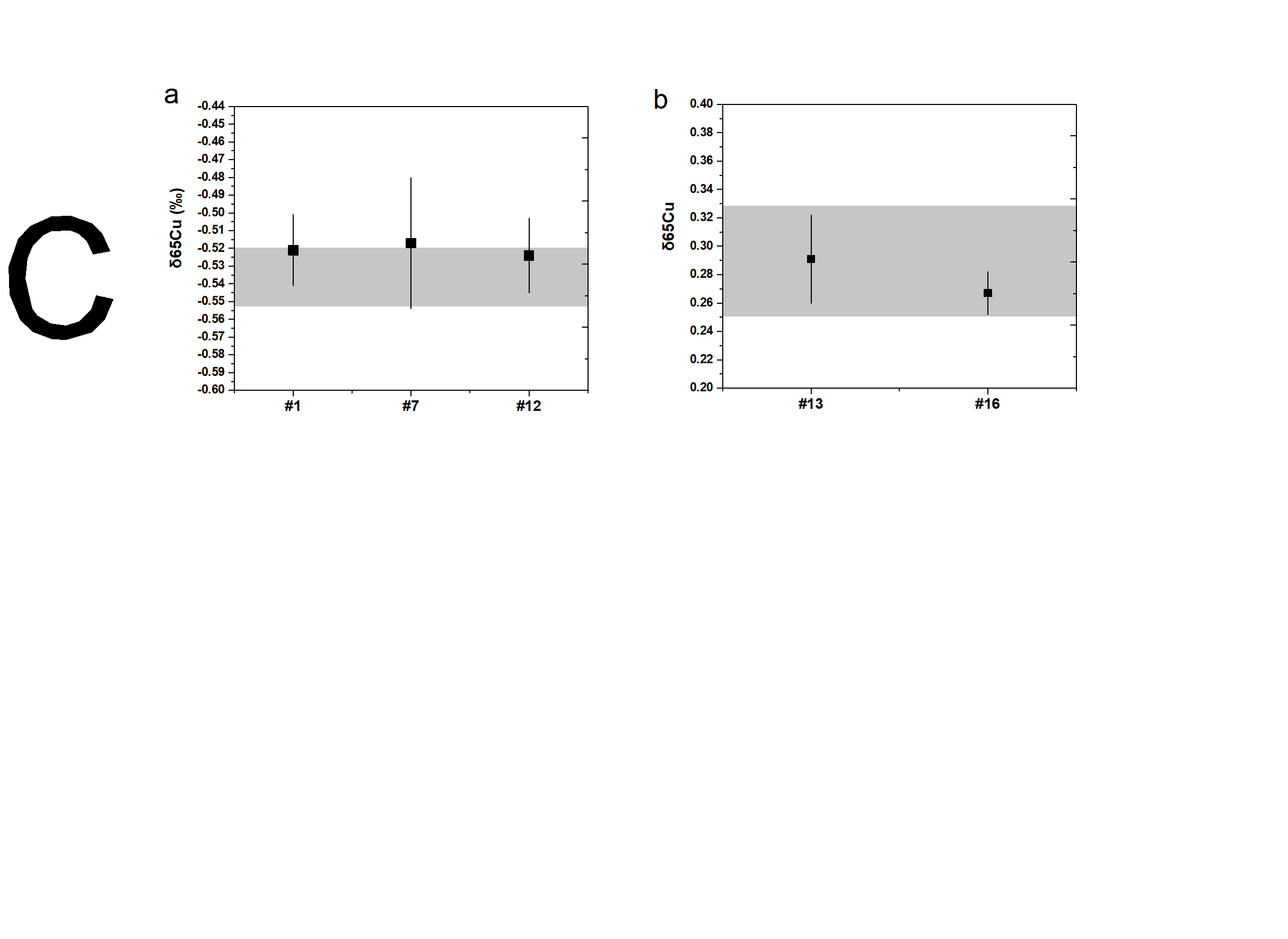
**

**Figure S2.** Cu isotopic composition for the recovered Au_95_Cu_5_ capsules in comparison with the initial Au_95_Cu_5_ capsules. (a) Wieland Edelwetalle, Germany. (b) Sino-Platinum Metals Corp. LTD., China. The grey areas represent the initial Cu isotopic composition before the experiments. The results show there is no change of Cu isotope compositions in the recovered capsules compared with the initial Au_95_Cu_5_ capsules.
